# Estimating prevalence of chronic obstructive pulmonary disease in the Southern Cone of Latin America: how different spirometric criteria may affect disease burden and health policies

**DOI:** 10.1186/s12890-017-0537-9

**Published:** 2017-12-11

**Authors:** Edgardo Sobrino, Vilma E. Irazola, Laura Gutierrez, Chung-Shiuan Chen, Fernando Lanas, Matías Calandrelli, Jacqueline Ponzo, Nora Mores, Pamela Serón, Allison Lee, Jiang He, Adolfo L. Rubinstein

**Affiliations:** 1 Institute for Clinical Effectiveness and Health Policy, Buenos Aires, Argentina; 2 Department of Epidemiology, Tulane University School of Public Health and Tropical Medicine, New Orleans, LA USA; 3 CIGES, Faculty of Medicine of the Universidad de la Frontera, Temuco, Chile; 4 Sanatorio San Carlos, Bariloche, Argentina; 5 Department of Family and Community Medicine, Universidad de la República, Montevideo, Uruguay; 6 Secretaría de Salud, Municipalidad de Marcos Paz, Pcia. de Buenos Aires, Argentina; 7 Icahn School of Medicine at Mount Sinai, Division of Pulmonary, Critical Care and Sleep Medicine, New York, NY USA; 8 National Ministry of Health, Buenos Aires, Argentina

**Keywords:** Chronic obstructive pulmonary disease, Prevalence, Fixed ratio, Lower limit normal, Risk factors, South America, Cross sectional study

## Abstract

**Background:**

Chronic obstructive pulmonary disease (COPD) is the fourth leading cause of death worldwide. The study aimed to determine and compare the prevalence of COPD in the general population aged 45-74 years old according to fixed ratio and lower limit of normal (LLN) thresholds in four cities in the Southern Cone of Latin America.

**Methods:**

The Pulmonary Risk in South America (PRISA) study used a 4-stage stratified sampling method to select 5814 participants from 4 cities in the Southern Cone of Latin America (Bariloche and Marcos Paz, Argentina; Temuco, Chile; and Pando-Barros Blancos, Uruguay). Data on demographic information, medical history, risk factors, pre-bronchodilator and post-bronchodilator spirometry were obtained using a standard protocol. According to GOLD, COPD was defined as a post-bronchodilator ratio of forced expiratory volume in one second (FEV1) over forced vital capacity (FVC) less than 70%. The LLN threshold was defined as the lower fifth percentile for predicted FEV1/FVC, and was evaluated as an alternative COPD definition.

**Results:**

Overall COPD prevalence was 9.3% (95% CI 8.4, 10.2%), and men had a higher prevalence [11.8% (95% CI 10.3, 13.3%)] than women [7.3% (95% CI 6.2, 8.3%)] with the fixed ratio. Overall COPD prevalence using LLN was 4.7% (95% CI 4.1, 5.3%), higher in men: 5.8% (95% CI 4.7, 6.8%) than women: 3.9% (95% CI 3.1, 4.7%). COPD prevalence was significantly higher among those who were older, had <high-school education and lower body-mass index, were cigarette smokers, and had self-reported history of asthma and tuberculosis.

**Conclusions:**

First**,** COPD and its risk factors are highly prevalent in the general population of Argentina, Chile, and Uruguay. Second, the prevalence of COPD by LLN criterion was significantly lower with lesser degrees of severity compared to fixed ratio of FEV1/FVC. Implementing LLN criterion instead of fixed ratio of FEV1/FVC may reduce the risk of over-diagnosis of COPD, although further prognostic studies of COPD adverse outcomes should be conducted using both definitions. Third, these data suggest that national efforts on the prevention, treatment, and control of COPD should be a public health priority in the Southern Cone of Latin America.

**Electronic supplementary material:**

The online version of this article (10.1186/s12890-017-0537-9) contains supplementary material, which is available to authorized users.

## Background

Chronic obstructive pulmonary disease (COPD) is the fourth leading cause of mortality worldwide [1, 2]. Under-diagnosis and a lack of rigorous population-based studies from low- and middle-income countries (LMIC) likely contribute to the underestimates of the actual COPD burden globally [3]. To date, only four population-based studies have been conducted in Latin America. They found a wide variation in COPD prevalence among populations [4–7]. The four studies used reliable measurements and consistent diagnostic criteria. Additionally, PLATINO and PREPOCOL studies were included in a recent study to explore the determinants of COPD prevalence worldwide, focused on its underdiagnosis, from international surveys [8].

There is a large agreement that the diagnostic confirmation of COPD requires spirometric evidence of post-bronchodilator airflow limitation that is not fully reversible. However, no consensus has been achieved regarding the cut-off for separating obstructed from healthy subjects. The Global Initiative for Chronic Obstructive Lung disease (GOLD) criteria defines COPD with a fixed ratio of post-bronchodilator forced expiratory volume in the first second to forced vital capacity (FEV1/FVC) of <0.7. However, the American Thoracic Society and the European Respiratory Society recommends setting the cut-off at the 5th percentile of the normal distribution (LLN), in order to avoid potential misclassification. This discrepancy can be translated into different estimates of the COPD prevalence and disease burden.

COPD is a progressive disease that is highly underdiagnosed, particularly among people with early stage disease, who could benefit from preventive strategies. Therefore, achieving accurate estimates of the prevalence of COPD is essential [9].

Here, we will report the population-based cross-sectional baseline results of the PRISA study, which aimed to determine and compare the prevalence of COPD according to fixed ratio and LLN thresholds. As a secondary aim, we analyze the strength of the associations between COPD and risk factors for each of the COPD definitions, such as age, sex, smoking history and education, as well as others risk factors for persistent post-bronchodilator obstruction, such as indoor air pollution from burning biomass fuels, asthma and tuberculosis.

## Methods

### Study participants

The details of study design and the sampling method of the PRISA study have been previously published [10]. Briefly, the Pulmonary Risk in South America (PRISA) study is a population-based prospective cohort study that evaluates the prevalence, incidence and determinants of COPD in the Southern Cone of Latin America as well as individual changes in lung function over time. A total of 5814 women and men aged 45 to 74 were recruited between February 2011 and December 2012 from randomly selected samples in four small to mid-sized cities in the Southern Cone of Latin America: two in Argentina (Marcos Paz and Bariloche), one in Chile (Temuco), and one in Uruguay (Pando-Barros Blancos). Marcos Paz and Pando-Barros Blancos are small cities with 54,000 and 58,000 residents, respectively, according to the latest census data of each country. Participants were recruited from the urban population of these sites. Bariloche (Argentina) and Temuco (Chile) are larger cities with 134,000 and 245,000 residents, respectively, according to the latest census of each country. These study locations were selected based on population characteristics reflecting their country averages.

A 4-stage stratified sampling method was used to select a representative sample of the general population of the Southern Cone of Latin America. In the first stage, census radii were randomly selected from each of the four locations, and stratified by socio-economic level. In the second stage, a number of blocks proportional to the radius size were randomly selected. In the third stage, households within each block were selected by systematic random sampling. All members in the selected households between 45 and 74 years of age were listed to create the study sampling frame. In the final stage of sampling, one listed member per household was randomly selected to be included in the study.

Male and female permanent residents aged 45-74 years living at one of the study locations for at least six months a year who were willing to participate in the study were eligible. Individuals with contraindications to spirometry testing (active tuberculosis; pregnancy; history of detached retina or myocardial infarction; and ocular, thoracic, or abdominal surgery within prior six weeks) were excluded. Among 5814 participants, 491 were ineligible or declined spirometry and 391 declined post-bronchodilator spirometry. In addition, participants with spirometric data which did not meet the American Thoracic Society/European Respiratory Society (ATS/ERS) standards were contacted to repeat the spirometry test.

### Data collection

Study data were collected at a home visit and a clinical visit. During the home visit, information on socio-demographic characteristics (age, sex, education, occupation, and household income), medical history (asthma, tuberculosis, COPD, and hospitalization due to pulmonary disease), and risk factors (tobacco use, secondhand smoking, indoor air pollution, and lifestyle factors) was obtained using a standard questionnaire. The Global Adult Tobacco Survey (GATS) questionnaire was used to assess former and current tobacco use, number of pack-years smoked, and secondhand smoking exposure [11]. Participants were defined as current smokers if they were smoking at the time of the survey and had smoked more than 100 cigarettes in their lifetime, and classified as former smokers if they smoked more than 100 cigarettes in their lifetime but were not current smokers [12]. Pack-years were calculated by multiplying the number of packs of cigarettes smoked per day by the number of years smoking. Participants were considered to have secondhand smoke exposure if they answered that someone had smoked in their presence at home and/or work for at least five of the past seven days [13]. Exposure to biomass was assessed by asking participants if they used wood, coal, or others biomass fuels to cook and heat their home and time of exposure (hours per day, days per week, and number of years). Exposure to biomass fuels was defined as ≥200 h/year [14–16].

Body weight and height were measured twice during the clinical examination. Weight was measured in light indoor clothing without shoes in kilograms to one decimal place, using standing scales supported on a steady surface. Height was measured without shoes in centimeters to one decimal place with a stadiometer. Body-mass index (BMI) was calculated as weight in kg/height in m^2^.

Spirometry was performed with a portable, battery operated, ultrasound transit-time based EasyOne™ spirometer (Medical Technologies, Chelmsford, Massachusetts and Zürich, Switzerland) [17]. Daily calibration was performed with a three-liter syringe. Trained professionals administered a questionnaire to determine spirometer eligibility and then performed spirometry following ATS/ERS guidelines [18]. Participants performed at least three and up to eight forced expiratory maneuvers until the forced vital capacity (FVC) and forced expiratory volume in the first second (FEV1) were reproducible within 150 mL. A beta-agonist bronchodilator (Albuterol 200 μg) was then administered and spirometry was repeated 15 min later, using the same criteria. All spirometric maneuvers were performed with the participant in a seated position, wearing a nose clip and using a disposable mouthpiece. Test results were stored in the spirometer and downloaded weekly to a central computer.

### Definitions

COPD was defined as a post-bronchodilator ratio of forced expiratory volume in one second (FEV1) over forced vital capacity (FVC) less than 0.7, according to the Global Initiative for Obstructive Lung Disease (GOLD) criteria [19]. The observed/predicted spirometry values based on the Perez Padilla reference equations for Latin America were calculated [20]. The lower limit of normal (LLN) threshold was evaluated as an alternative COPD definition. The LLN was defined as the lower fifth percentile for predicted FEV1/FVC. The reduction in FEV1 was used to stage the degree of obstruction: GOLD stage I was defined as FEV1 ≥ 80% predicted, GOLD stage II as FEV1 50-79% predicted, GOLD stage III as FEV1 30-49% predicted, and GOLD stage IV as FEV1 < 30% predicted.

### Statistical analysis

The PRISA study was designed to provide precise estimates of the prevalence of COPD by sex and region (Marcos Paz and Bariloche, Argentina; Temuco, Chile; and Pando-Barros Blancos, Uruguay) in three age groups: 45-54, 55-64, and 65-74 years old. Sample sizes were estimated to meet recommended requirements for precision in a complex survey [21]. All calculations were weighted to represent the general adult population aged 45-74 years in the study sites. Weights were calculated using data from the 2010 Population Census and the PRISA study sampling scheme, and took into account several features of the survey, including oversampling for specific age groups, non-response, and other demographic differences between the sample and the total population.

Mean level and prevalence estimates of COPD were calculated for the overall population and by the three age groups. Additionally, age-standardized prevalence estimates were calculated for men and women, and the four study sites, after age-standardization to the overall 2010 population distribution in the Southern Cone of Latin America. Standard errors were calculated by a technique appropriate for the complex survey design. Multivariable logistic regression analyses were conducted to explore risk factors for COPD in all study participants, as well as in current smokers, non-current smokers, and never smokers. Using the LLN definition as the reference standard to identify true obstruction, we calculated the agreement between both criteria using the kappa estimate. We also calculated sensitivity, specificity, positive predicted value, and negative predicted value of the fixed ratio. All data analyses were generated using SAS/STAT software, Version 9.3 of the SAS System for Windows (SAS Institute Inc., Cary, NC, USA) and STATA 12.0 (StataCorp LP, College Station, TX, USA).

The study complies with the Declaration of Helsinki. The study protocol has been approved by the Institutional Review Boards in all participating institutes in Argentina, Chile, Uruguay, and the US. The written informed consent has been obtained from all study participants. All participants received test result feedback.

## Results

**Fig. 1:**
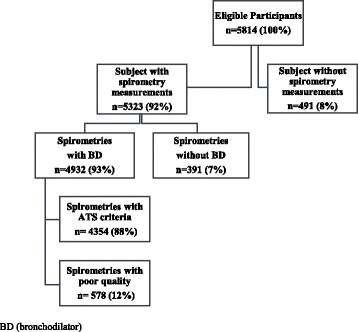
Flowchart showing study participants

From 5814 eligible participants, spirometry was not performed in 491, was not repeated with bronchodilators in 391, and was considered of poor quality in 578 participants (Fig. 1).

Final analyses were based on 4354 PRISA study participants, (74.9% of the overall sample). Additional file 1: Table S1 shows the percentage of non-respondents and excluded participants according to location, sex, age by groups and education. Non respondents were slightly higher among women, older age, higher education, and quite similar between smokers and nonsmokers. The general characteristics of the study population aged 45-74 in the four cities of the Southern Cone of Latin America are presented in Table 1. Overall, approximately 27.3% (CI 95% 25.7, 28.9) of the general population was current smokers. The prevalence of smoking was significantly higher in men, 30.9% (CI 95% 28.3, 33.4) than in women, 24.3% (CI 95% 22.2, 26.3). Likewise, overall 28.7% (CI 95% 27.1, 30.3) were former smokers, with a prevalence of 36.5% (CI 95% 34.0, 39.1) in men and 22.2% (CI 95% 20.3, 24.1) in women. Among current smokers, 25.4% (CI 95% 22.0, 28.7) smoked 10-19 pack-years in lifetime and 47.4% (CI 95% 43.6, 51.2) smoked more than 20 pack-years in lifetime. Approximately 18.7% (CI 95% 17.4, 20.0) of the general population was exposed to second-hand smoking (19.9% in men and 17.7% in women) and 35.0% (CI 95% 33.3, 36.7) were exposed to burning biomass fuels (30.5% in men and 38.7% in women). In addition, 6.0% (CI 95% 5.2, 6.8) of the population had a self-reported history of asthma (4.3% in men and 7.4% in women) and 1.6% (CI 95% 1.2, 2.1) self-reported having tuberculosis (1.5% in men and 1.8% in women).

**Table 1:** General Characteristics and Risk Factors of the Study Population in the Southern Cone of Latin America

	No. of study participants	<high school, %	Body-mass index (mean, kg/m^2^)	Cigarette smoking, %		Pack-year in current smokers, %		Second-hand smoking,^a^ %	Biomass exposure,^a^ %	Self-reported asthma, %	Self-reported tuberculosis, %
				Current smoker	Former smoker	10-19 pack-year	≥20 pack-year				
Overall	4354	58.8 (57.1, 60.6)	28.9 (28.7, 29.0)	27.3 (25.7, 28.9)	28.7 (27.1, 30.3)	25.4 (22.0, 28.7)	47.4 (43.6, 51.2)	18.7 (17.4, 20.0)	35.0 (33.3, 36.7)	6.0 (5.2, 6.8)	1.6 (1.2, 2.1)
Sex											
Men	1790	58.7 (56.1, 61.4)	28.5 (28.3, 28.7)	30.9 (28.4, 33.4)	36.5 (34.0, 39.1)	23.2 (18.4, 27.9)	56.8 (51.3, 62.3)	19.9 (17.8, 21.9)	30.5 (28.0, 33.0)	4.3 (3.3, 5.3)	1.5 (0.8, 2.1)
Women	2564	58.9 (56.5, 61.2)	29.2 (29.0, 29.4)	24.3 (22.2, 26.3)	22.2 (20.3, 24.1)	27.8 (23.1, 32.5)	37.0 (32.3, 41.7)	17.7 (16.0, 19.4)	38.7 (36.4, 41.0)	7.4 (6.1, 8.6)	1.8 (1.2, 2.4)
Location											
Marcos Paz, Argentina	1245	80.5 (78.2, 82.8)	30.0 (29.7, 30.2)	28.6 (25.9, 31.2)	26.0 (23.5, 28.6)	20.6 (15.6, 25.6)	71.9 (66.3, 77.4)	30.2 (27.5, 32.9)	4.7 (3.4, 5.9)	5.2 (4.0, 6.5)	1.4 (0.7, 2.0)
Bariloche, Argentina	1107	65.9 (63.0, 68.8)	28.4 (28.1, 28.6)	28.0 (25.2, 30.9)	31.5 (28.7, 34.4)	24.6 (19.3, 30.0)	51.3 (44.9, 57.6)	19.1 (16.6, 21.5)	57.8 (54.7, 60.8)	4.6 (3.4, 5.9)	1.3 (0.7, 2.0)
Temuco, Chile	1063	42.1 (39.1, 45.2)	29.0 (28.8, 29.3)	26.1 (23.3, 29.0)	26.4 (23.7, 29.1)	30.6 (23.3, 37.9)	24.6 (18.0, 31.3)	12.0 (9.9, 14.1)	37.4 (34.4, 40.4)	6.2 (4.7, 7.7)	1.9 (1.1, 2.7)
Barros Blancos, Uruguay	939	76.3 (73.5, 79.1)	28.7 (28.4, 29.0)	28.1 (25.0, 31.1)	31.6 (28.6, 34.7)	20.4 (14.7, 26.1)	65.6 (58.9, 72.2)	27.7 (24.7, 30.7)	11.2 (9.1, 13.2)	8.0 (6.3, 9.8)	1.6 (0.8, 2.5)
Age groups, years											
45-54	1610	51.9 (49.1, 54.7)	28.5 (28.2, 28.8)	36.2 (33.5, 39.0)	24.1 (21.7, 26.5)	26.9 (22.3, 31.4)	43.5 (38.5, 48.5)	23.5 (21.2, 25.7)	30.9 (28.2, 33.5)	5.7 (4.4, 6.9)	1.2 (0.6, 1.7)
55-64	1594	61.6 (58.8, 64.4)	28.8 (28.5, 29.0)	23.1 (20.8, 25.5)	33.7 (31.1, 36.4)	21.2 (15.9, 26.6)	52.8 (46.4, 59.1)	16.4 (14.5, 18.4)	37.7 (35.0, 40.5)	6.2 (4.8, 7.6)	2.2 (1.3, 3.0)
65-74	1150	71.2 (68.2, 74.2)	29.4 (29.2, 29.7)	12.1 (10.0, 14.2)	31.6 (28.6, 34.6)	28.4 (18.5, 38.2)	58.8 (48.2, 69.3)	10.7 (8.9, 12.5)	40.5 (37.2, 43.7)	6.4 (4.9, 8.0)	1.9 (0.9, 2.9)

Data are percentages (95% CI)

^a^Second-hand smoking was defined as exposed to second-hand smoking ≥5 days/week at home or work; biomass exposure was defined as the use of wood or coal for cooking ≥200 times/year

### Measures of pulmonary function

Pulmonary function measures can be seen in Table 2. Overall mean pre-bronchodilator FEV1 was 2.58 L (CI95% 2.56, 2.61) and pre-bronchodilator FVC was 3.35 L (CI 95% 3.33, 3.38) with a pre-bronchodilator FEV1/FVC ratio of 76.8% (CI 95% 76.6, 77.1). Pre-bronchodilator FEV1 and FVC were significantly higher in men than in women, but FEV1/FVC were higher in women, and decreased with aging. Overall mean post-bronchodilator FEV1 was 2.65 L and post-bronchodilator FVC was 3.37 L with a post-bronchodilator FEV1/FVC ratio of 78.5%. Post-bronchodilator FEV1 and FVC were significantly higher in men but FEV1/FVC ratio was higher in women. Post-bronchodilator FEV1, FVC, and FEV1/FVC ratio all decreased with aging.

**Table 2:** Age-adjusted and Age-specific Pulmonary Function Measures of the Study Population in the Southern Cone of Latin America

	FEV_1_,^a^		FVC^a^		FEV_1_/FVC ratio	
	Pre-BD	Post-BD	Pre-BD	Post-BD	Pre-BD	Post-BD
Overall	2.58 (2.56, 2.61)	2.65 (2.63, 2.67)	3.35 (3.33, 3.38)	3.37 (3.34, 3.40)	76.84 (76.63, 77.06)	78.51 (78.29, 78.73)
Men	3.04 (3.01, 3.07)	3.12 (3.09, 3.15)	3.98 (3.95, 4.02)	4.01 (3.97, 4.04)	75.99 (75.64, 76.34)	77.56 (77.20, 77.91)
45-54 years	3.39 (3.34, 3.44)	3.46 (3.41, 3.51)	4.34 (4.28, 4.40)	4.34 (4.29, 4.40)	78.05 (77.58, 78.52)	79.76 (79.28, 80.23)
55-64 years	2.92 (2.87, 2.98)	3.00 (2.95, 3.06)	3.86 (3.80, 3.92)	3.88 (3.82, 3.94)	75.37 (74.70, 76.03)	77.04 (76.36, 77.73)
65-74 years	2.53 (2.47, 2.59)	2.62 (2.57, 2.68)	3.46 (3.40, 3.53)	3.54 (3.47, 3.60)	72.85 (72.10, 73.61)	74.00 (73.24, 74.75)
Women	2.21 (2.19, 2.23)	2.26 (2.24, 2.28)	2.84 (2.81, 2.86)	2.84 (2.82, 2.87)	77.53 (77.26, 77.81)	79.28 (79.00, 79.55)
45-54 years	2.46 (2.43, 2.50)	2.52 (2.49, 2.55)	3.12 (3.09, 3.16)	3.12 (3.08, 3.15)	78.85 (78.45, 79.26)	80.68 (80.25, 81.11)
55-64 years	2.13 (2.10, 2.17)	2.19 (2.15, 2.22)	2.75 (2.72, 2.79)	2.76 (2.73, 2.80)	77.29 (76.83, 77.74)	78.94 (78.51, 79.37)
65-74 years	1.81 (1.78, 1.84)	1.87 (1.83, 1.90)	2.40 (2.36, 2.44)	2.42 (2.38, 2.46)	75.31 (74.69, 75.93)	77.02 (76.43, 77.60)

Data are means (95% CI)

^a^ FEV1 = forced expiratory volume in one second (liters) and FVC = forced vital capacity

### Prevalence of COPD

The overall prevalence of spirometric airflow limitation was 9.3% (CI 95% 8.4, 10.2) by GOLD criteria using the fixed ratio (FR), and 4.7% (CI 95% 4.1, 5.3) by LLN in the general population aged 45-74 years in the Southern Cone of Latin America (Table 3). The prevalence of airflow limitation by fixed ratio was greater than that by LLN, except among young women where both estimates were similar: 4.1% (CI 95% 2.7, 5.4) vs 3.5% (CI 95% 2.2, 4.8) with confidence intervals with overlapping values. Larger differences in prevalence between both criteria were seen among men as compared to women, and these differences increased with aging. In the older age group (65­74) the difference in prevalence estimates between the two diagnostic criteria more than doubled: 23.3% FR (CI 95% 19.3, 27.3) vs 9.9% LLN (CI 95% 7.2,12.6) in men but in age group this discrepancy was also seen in women. 13.8% FR (CI 95% 11.0, 16.6) vs 4.8% LLN (CI 95% 3.1, 6.4).

**Table 3:** Age-standardized and Age-specific Prevalence of Chronic Obstructive Pulmonary Disease According to GOLD and Lower Limit Normal Stage in the Southern Cone of Latin America

	COPD according to FEV_1_/FVC				COPD according to LLN			
	Total	Stage I	Stage II	Stage III-IV	Total	Stage I	Stage II	Stage III-IV
Overall	9.3 (8.4, 10.2)	3.7 (3.1, 4.3)	4.2 (3.6, 4.8)	1.4 (1.0, 1.8)	4.7 (4.1, 5.3)	0.9 (0.6, 1.2)	2.5 (2.0, 2.9)	1.3 (1.0, 1.7)
Men	11.8 (10.3, 13.3)	5.0 (4.0, 6.1)	5.1 (4.1, 6.1)	1.7 (1.1, 2.3)	5.8 (4.7, 6.8)	1.0 (0.5, 1.5)	3.1 (2.3, 3.9)	1.6 (1.0, 2.2)
45-54 years	5.5 (3.7, 7.4)	2.9 (1.5, 4.4)	2.1 (1.0, 3.1)	0.5 (0.0, 1.0)	3.4 (2.0, 4.7)	0.9 (0.2, 1.6)	2.0 (0.9, 3.0)	0.5 (0.0, 1.0)
55-64 years	12.3 (9.7, 15.0)	4.9 (3.1, 6.6)	4.6 (2.9, 6.2)	2.9 (1.5, 4.3)	6.2 (4.2, 8.1)	1.1 (0.2, 1.9)	2.2 (1.1, 3.3)	2.9 (1.5, 4.3)
65-74 years	23.3 (19.3, 27.3)	9.5 (6.7, 12.2)	11.7 (8.7, 14.7)	2.1 (0.8, 3.5)	9.9 (7.2, 12.6)	1.2 (0.2, 2.2)	6.7 (4.5, 8.9)	2.0 (0.7, 3.2)
Women	7.3 (6.2, 8.3)	2.6 (2.0, 3.2)	3.5 (2.8, 4.2)	1.2 (0.7, 1.7)	3.9 (3.1, 4.7)	0.7 (0.4, 1.1)	2.0 (1.5, 2.6)	1.1 (0.6, 1.6)
45-54 years	4.1 (2.7, 5.4)	1.1 (0.5, 1.8)	2.0 (1.1, 2.9)	1.0 (0.2, 1.8)	3.5 (2.2, 4.8)	0.9 (0.3, 1.5)	1.7 (0.8, 2.5)	1.0 (0.2, 1.8)
55-64 years	7.1 (5.4, 8.8)	2.7 (1.6, 3.8)	3.4 (2.2, 4.6)	1.1 (0.5, 1.6)	3.7 (2.5, 4.9)	0.5 (0.1, 0.9)	2.2 (1.2, 3.1)	1.1 (0.5, 1.6)
65-74 years	13.8 (11.0, 16.6)	5.4 (3.5, 7.4)	6.5 (4.6, 8.3)	1.9 (0.7, 3.0)	4.8 (3.1, 6.4)	0.9 (0.1, 1.6)	2.4 (1.3, 3.5)	1.5 (0.5, 2.5)
Location								
Marcos Paz, Argentina	17.1 (15.0, 19.1)	6.9 (5.5, 8.3)	8.3 (6.8, 9.8)	1.8 (1.1, 2.5)	9.7 (8.1, 11.4)	1.7 (0.9, 2.4)	6.1 (4.8, 7.4)	1.8 (1.1, 2.5)
Bariloche, Argentina	9.9 (8.1, 11.6)	4.4 (3.2, 5.7)	4.4 (3.2, 5.7)	1.0 (0.4, 1.5)	4.3 (3.1, 5.5)	1.1 (0.5, 1.7)	2.2 (1.3, 3.2)	1.0 (0.4, 1.5)
Temuco, Chile	6.4 (5.0, 7.8)	2.9 (1.9, 3.9)	2.2 (1.4, 3.0)	1.3 (0.6, 1.9)	2.9 (1.9, 3.9)	0.5 (0.1, 1.0)	1.2 (0.6, 1.8)	1.2 (0.6, 1.8)
Barros Blancos, Uruguay	11.0 (9.0, 13.0)	2.7 (1.7, 3.7)	6.2 (4.7, 7.7)	2.1 (1.2, 3.1)	6.7 (5.1, 8.4)	0.9 (0.2, 1.5)	3.9 (2.7, 5.2)	2.0 (1.0, 2.9)

Data are percentages (95% CI)

The prevalence of COPD was significantly higher in men (11.8% FR (CI 95% 10.3, 13.3) or 5.8% LLN (CI 95% 4.7, 6.8)) than in women (7.3% FR (CI 95% 6.2, 8.3) or 3.9% LLN (CI 95% 3.1, 4.7)), and rapidly increased with age using both diagnostic criteria. The ratio of forced expiratory volume in the first second to forced vital capacity (FEV1/FVC) by age in adult men and women are shown in Additional file 2: Figure S1 and Additional file 3: Figure S2.

The prevalence of stages I, II, and III-IV COPD was 3.7%, 4.2% and 1.4%, respectively with the FR, and 0.9%, 2.5% and 1.3%, respectively with LLN. As was expected, stages III and IV were similar using both criteria, and differences in overall prevalence between both methods were mostly explained by a higher prevalence of less severe stages (I and II) in GOLD compared to LLN (Table 3).

Overall agreement between LLN and FR was moderate, kappa coefficient = 0.66 (95% CI 0.65, 0.67), though better in participants between 45 and 54 years old (kappa coefficient = 0.84). Overall, sensitivity, specificity, positive predictive value, and negative predictive value of the fixed ratio were 100%, 95.5%, 51.6% and 100%, respectively (Additional file 4: Table S2).

### Risk factors for COPD

Age-standardized prevalence of COPD was significantly higher in individuals with less than high-school education, BMI < 25 kg/m^2^, current cigarette smoking, exposure to second-hand smoking, and a self-reported history of asthma or tuberculosis, irrespective of the criteria used to define COPD (Additional file 5: Table S3). Among current smokers, there was a dose-response association between lifetime pack-years of smoking and prevalence of COPD. We also analyzed the relationship between intensity, duration of smoking, and prevalence of COPD. Both factors were significantly associated with this condition (Additional file 6: Table S4). In the logistic regression analysis adjusted for age, sex, and study locations (model 1), male sex, older age, <high-school education, BMI < 25 kg/m^2^, current cigarette smoking, pack-years, history of asthma, and history of tuberculosis were significantly associated with increased risk of COPD (Table 4). In model 2, which simultaneously included all risk factors in the model 1 (except cigarette smoking or second-hand smoking), male sex, older age, BMI < 25 kg/m^2,^ pack-years smoked, history of asthma and history of tuberculosis were significantly associated with increased risk of COPD.

**Table 4:** Odds Ratios (95% Confidence Intervals) for Chronic Obstructive Pulmonary Disease among Adults Aged 45-74 Years in the Southern Cone of Latin America

Risk factors		COPD according FEV_1_/FVC Odds ratios and 95% CI		COPD according LLN Odds ratios and 95% CI	
		Model 1	Model 2	Model 1	Model 2
Sex, men		1.7 (1.4, 2.1)	1.8 (1.4, 2.3)	1.5 (1.1, 2.0)	1.5 (1.1, 2.2)
Age, years	45-54	ref	ref	ref	ref
	55-64	2.1 (1.5, 2.9)	2.3 (1.7, 3.3)	1.4 (1.0, 2.0)	1.5 (1.0, 2.2)
	65-74	4.6 (3.4, 6.2)	6.1 (4.4, 8.6)	2.1 (1.4, 3.0)	2.7 (1.7, 4.1)
< High-school education		1.4 (1.1, 1.8)	1.5 (1.1, 2.0)	1.7 (1.2, 2.5)	1.8 (1.1, 2.7)
Body-mass index <25 kg/m^2^		1.7 (1.3, 2.1)	1.4 (1.1, 1.9)	2.2 (1.6, 3.0)	1.9 (1.3, 2.7)
Cigarette smoking	Never	ref	ref	ref	ref
	Former	1.3 (0.9, 1.7)	1.1 (0.8, 1.4)	1.4 (1.0, 2.2)	1.2 (0.7, 1.8)
	Current	2.8 (2.1, 3.7)	2.8 (2.1, 3.8)	3.1 (2.1, 4.6)	3.1 (2.1, 4.5)
Lifetime exposure in current smokers, pack-years	<10	ref	–	ref	–
	10-19	2.1 (1.0, 4.4)	–	1.0 (0.4, 2.7)	–
	≥20	4.0 (2.1, 7.7)	–	2.2 (1.1, 4.4)	–
Second-hand smoking		1.0 (0.6, 1.6)	–	0.8 (0.4, 1.4)	–
Exposure to biomass		1.2 (0.9, 1.6)	1.2 (0.8, 1.7)	1.3 (0.8, 2.1)	1.2 (0.7, 2.0)
Self-reported history of asthma		8.8 (6.2, 12.5)	9.8 (6.7, 14.4)	10.8 (7.3, 16.0)	13.0 (8.4, 19.9)
Self-reported history of tuberculosis		3.9 (1.8, 8.3)	3.3 (1.4, 7.8)	6.4 (2.9, 14.2)	5.6 (2.2, 14.3)

Model 1: model for each risk factor adjusted for age, sex, and study site; Model 2: model adjusted for age, sex, study site, high school education, body-mass index, cigarette smoking, exposure to biomass, self-reported history of asthma, and self-reported history of tuberculosis

## Discussion

We are reporting here the prevalence and risk factors of COPD in the general population of the Southern Cone of Latin America. PRISA utilized a multistage sampling method to select a representative sample of the general population aged 45-74. In addition, standardized methods consistent with ATS/ERS guidelines were used to collect spirometry data. Therefore, our results are comparable with those from other international studies.

Our data documented that, while COPD is highly prevalent in the Southern Cone of Latin America, specific prevalence estimates are dependent on the criteria used to define COPD. We found an estimated prevalence of 9.3% using GOLD fixed ratio and 4.7% using ATS lower limit of normal. With both criteria, the prevalence of COPD was higher in men than in women, related to historical smoking patterns, and increased with aging [22]. Low education, body-mass index, current cigarette smoking and history of asthma or tuberculosis, were associated with an increased prevalence of COPD or at least with persistent post-bronchodilator obstruction regardless of the diagnostic criteria used.

COPD population-based prevalence estimates comparisons across different geographic locations are generally based on GOLD criteria (fixed ratio) as population studies in the region have mainly used this diagnostic criterion to define COPD. In this regard, the prevalence of COPD was lower in our study compared to that reported in the PLATINO study [5]. However, our data show a higher prevalence of GOLD stage II, III and IV than that reported by PLATINO. Since pulmonary volumes, especially FEV1, decline with age, the use of a fixed ratio of FEV1/FVC to define COPD may result in over-diagnosis of this condition in older populations [23]. This was clearly observed in our study where the differences in COPD prevalence estimates between the two sets of diagnostic criteria were much larger in older age groups than in younger age groups, independent of sex. In this regard, the lack of a widespread and accepted diagnostic criteria of COPD may lead to a potential problem of over-diagnosis. There is currently no consensus on the best criteria to be used for the spirometric confirmation of clinical diagnosis of COPD. A debate revolves around the two airflow limitation definitions used, either a fixed ratio with a FEV1 less than 80% of the predicted value (GOLD II, III, and IV) or LLN. However, in advanced stages, where spirometric criteria for airflow limitation are associated with more symptoms and frequency of exacerbations, both methods show similar prevalence rates [24].

A reference standard for diagnosing COPD is currently lacking, particularly when the relationship between diagnostic criteria and clinical relevance is evaluated. The association of the two criteria with clinical outcomes has been assessed in a recent systematic review [25]. This review, based on 11 studies, showed that both the FR and LLN criteria are related with clinically relevant outcomes but LLN tends to better reflect FEV1-decline whereas FR might be better associated with comorbidity. Other authors argue that disagreement between FR and LLN is suggestive for an alternative diagnosis and reconsidered to require both spirometric abnormalities to reduce overdiagnosis of COPD. Misdiagnosing patients might lead to poorer outcomes, inappropriate medication, or incorrect treatments, especially if misdiagnosing involves cardiovascular disease [26, 27].

The definition of airflow limitation still leads to controversy. The FR method may be heavily biased by age. There was a steady increase in false positive results with advancing age; false positives exceeded 10% in subjects 64 years old in our study.

On the other hand, risk factors and strength of associations with COPD were similar using either GOLD or LLN criteria. In this regard, cigarette smoking continues to be a major public health challenge in the Southern Cone of Latin America [28]. In our study, current smoking is strongly and independently associated with increased risk of COPD. In addition, we identified a dose-response association between lifetime pack-years of smoking and prevalence of COPD among current smokers. We did not find any differences between intensity of tobacco use vs duration as a risk factor for COPD. There was no significant difference in COPD prevalence between former smokers and never smokers after adjustment for multiple risk factors. These results indicate that smoking-related risk of COPD could be eradicated after smoking cessation.

In our study, mild to moderate COPD was associated with overweight while lower BMI was related to more severe stages using both methods to estimate COPD prevalence. Several previous studies have reported that the prevalence of COPD is higher in those with lower BMI. Nutritional depletion and weight loss is common among patients with COPD, most likely as a consequence of COPD. In addition, low BMI is associated with poor prognosis and higher mortality in these patients [29–31].

Education level is an important index of socioeconomic status, and low socioeconomic status has been associated with increased risk of COPD as well as poor prognostic outcomes among patients with COPD [32, 33]. Our study found that less than high-school education was associated with higher prevalence of COPD, independently from other risk factors. Furthermore, this association was even stronger among never smokers. Self-reported history of asthma and tuberculosis was associated with persistent airflow limitation. Asthma is the strongest risk factor for airflow limitation in our study: a history of asthma was associated with an increase of COPD in both current smokers and never smokers. It was suggested that chronic airway inflammation and airflow obstruction in individuals with asthma and increased airway hyper-responsiveness might cause lung remodeling from thickening and fibrosis of the airway walls [34]. This remodeling process could result in irreversible and progressive airflow obstruction. In a cohort study of 3099 patients, asthma was the strongest risk factor for subsequent COPD, even more than tobacco smoking (hazard ratio 12.5 vs 2.9, attributable risk 18.5% vs 6.7%) [35].

Interestingly, in the PRISA study, among all the patients with diagnosis of COPD, only 14% received inhaled bronchodilators, and 87% of them used only short-acting β-agonists or short-acting muscarinic antagonists (SAMA). Inhaled corticosteroids (IC) alone or combined with long-acting bronchodilators were used by less than 10% of the population.

While asthma does not cause COPD, symptoms are similar, which may cause misclassification in studies which do not provide testing to differentiate asthma from COPD.

Tuberculosis was associated with an increase of COPD in never-smokers. Pulmonary tuberculosis is a specific infectious disease associated with airway fibrosis, and the immune response to mycobacteria can result in airway inflammation, which is characteristic of airways obstruction [36]. A positive association between self-reported history of asthma as a major risk factor for COPD and post-treatment pulmonary tuberculosis was found in all studies conducted in Latin America [37]. Well-designed prospective studies are necessary to assess direction of association.

Exposure to biomass fuel in this study was not associated with airway obstruction or COPD. Biomass fuel is associated with a chronic inflammatory response and ultimately pulmonary damage that increases the risk of COPD. We found a higher use of biomass fuels for cooking in two cities, Temuco in Chile (37.4%) and Bariloche in Argentina (57.8%), according to participant self-report. However, the study was conducted in an urban setting, and most of the population studied used clean cookstoves, which may explain this lack of association [38, 39].

Some limitations in our study should be mentioned. First, we did not use the NHANES III reference equation, which was the standard in the PLATINO and BOLD studies, two of the most important population based studies to estimate COPD prevalence. However, we used the reference equation of Perez Padilla for the Latin American population, that has been used in the PREPOCOL study [4, 20]. Second, we did not analyze respiratory symptoms. Recently, it has been remarked the importance of respiratory symptoms as factors at least as sensitive as airflow limitation in establishing a diagnosis of smoking induced disease. [40, 41] Third, exposure to biomass fuel was measured by a questionnaire, not by objective methods. Third, self-reported diagnostic of asthma or post-treatment tuberculosis, has limitations compared to direct diagnostic evaluation. Fourth, the sampling frame in each country is not nationally representative. While the study sample was randomly selected from each city included, caution is needed to extrapolate our findings to the overall country. However, socio-demographic data as well as risk factor prevalence of selected geographic locations are consistent with the results shown in national surveys in the Southern Cone, which suggests no major biases due to the selection of cities included in the PRISA study.

## Conclusions

The PRISA study, a large population-based study of the Southern Cone of Latin America, documented a large burden of COPD in the Southern Cone of Latin America. The prevalence of COPD by LLN criterion was significantly lower compared to GOLD criterion based on the fixed ratio of FEV1/FVC. This difference in prevalence rates was mostly explained by higher rates of stage I COPD according to GOLD. Estimating COPD prevalence by LLN criterion instead of fixed ratio of FEV1/FVC, or using both definitions may reduce the risk of over-diagnosis of COPD. Low education, low body-mass index, current cigarette smoking, and history of asthma or tuberculosis were associated with an increased prevalence of COPD, regardless of the diagnostic criteria used. While further prospective studies are needed to evaluate whether the discrepancies found between both methods may have implications for management and treatment with the aim of reducing the global burden of COPD, these data suggest that national efforts on the prevention, treatment, and control of COPD must be a public health priority in the Southern Cone of Latin America.

## Additional files

Additional file 1: Table S1. Percentage of non-respondents or excluded participants by location, sex, age, education and smoking status. (DOCX 15 kb)

Additional file 2: Figure S1. Ratio of forced expiratory volume in the first second to forced vital capacity (FEV1/FVC) versus age in adult men. (DOCX 167 kb)

Additional file 3: Figure S2. Ratio of forced expiratory volume in the first second to forced vital capacity (FEV1/FVC) versus age in adult women. (DOCX 213 kb)

Additional file 4: Table S2. Performance of the Fixed-Ratio method versus the Lower-Limit-of-Normal method. (DOCX 15 kb)

Additional file 5: Table S3. Age-standardized Prevalence (95% Confidence Intervals) of Chronic Obstructive Pulmonary Disease According to Demographic and Other Risk Factors. (DOCX 19 kb)

Additional file 6: Table S4. Age-standardized Prevalence (95% Confidence Intervals) of Chronic Obstructive Pulmonary Disease in Current Smokers according to intensity and duration exposure. (DOCX 14 kb)

## Abbreviations

ATS: American Thoracic Society
BMI: Body-mass index
BOLD: Burden of Obstructive Lung Disease Study
CI: Confidence Interval
COPD: Chronic obstructive pulmonary disease
ERS: European Respiratory Society
FEV1: Forced expiratory volume in 1 s
FR: Fixed ratio
FVC: Forced vital capacity
GATS: The Global Adult Tobacco Survey
GOLD: Global Initiative for Chronic Obstructive Lung disease
L: Litres
LLN: Lower limit of normal
LMIC: Middle-income countries
mL: Millilitres
NHANES: National Health and Nutrition Examination Survey
PLATINO: Latin American Project for Research in Pulmonary Obstruction
Post-BD: Post-bronchodilator
Pre-BD: Pre-bronchodilator
PREPOCOL: Prevalencia de EPOC en Colombia
PRISA: The Pulmonary Risk in South America Study
