# Supplementary material for: Estimating prevalence of chronic obstructive pulmonary disease in the Southern Cone of Latin America: how different spirometric criteria may affect disease burden and health policies
Source: BMC Pulm Med. 2017 Dec 11;17:187. doi: 10.1186/s12890-017-0537-9 (PMC5725644; doi:10.1186/s12890-017-0537-9)
Supplement: Supplementary file 1 — Percentage of non-respondents or excluded participants by location, sex, age, education and smoking status. (DOCX 15 kb) [file 12890_2017_537_MOESM1_ESM.docx]

Supplementary Table 1: Percentage of non-respondents or excluded participants by location, sex, age, education and smoking status

| Characteristics | |  | Marcos Paz, Argentina | Bariloche, Argentina | | Temuco, Chile | | Barros Blancos, Uruguay |  | |
| --- | --- | --- | --- | --- | --- | --- | --- | --- | --- | --- |
|  |  |  |  |  |  |  |  |  |  |  |
| Overall | | 18.7 | | 29.2 | | 28.1 | 24.0 | |  |  |
| Sex Men | | 16.9 | | 28.3 | | 25.7 | 22.9 | |  |  |
| Women | | 21.3 | | 30.5 | | 30.9 | 25.6 | |  |  |
| Age groups, years 45-54 | | 15.7 | | 23.5 | | 28.9 | 20.5 | |  |  |
| 55-64 | | 20.3 | | 29.6 | | 26.3 | 21.3 | |  |  |
| 65-74 | | 20.4 | | 38.0 | | 29.2 | 30.4 | |  |  |
| Education Level < High-school | | 17.5 | | 25.9 | | 27.1 | 22.8 | |  |  |
| ≥ High-school | | 19.0 | | 30.7 | | 29.3 | 24.3 | |  |  |
| Current Smoker No | | 19.4 | | 30.0 | | 27.5 | 23.2 | |  |  |
| Yes | | 16.5 | | 26.8 | | 30.0 | 26.2 | |  |  |
