# Supplementary material for: Estimating prevalence of chronic obstructive pulmonary disease in the Southern Cone of Latin America: how different spirometric criteria may affect disease burden and health policies
Source: BMC Pulm Med. 2017 Dec 11;17:187. doi: 10.1186/s12890-017-0537-9 (PMC5725644; doi:10.1186/s12890-017-0537-9)
Supplement: Supplementary file 3 — Ratio of forced expiratory volume in the first second to forced vital capacity (FEV1/FVC) versus age in adult women. (DOCX 213 kb) [file 12890_2017_537_MOESM3_ESM.docx]

Supplementary Figure 2: Ratio of forced expiratory volume in the first second to forced vital capacity (FEV1/FVC) versus age in adult women

Red line, prediction equation*; blue line, lower limit of normal

*Prediction equation derived from Perez Padilla reference for Latin America adult population
