# Supplementary material for: Estimating prevalence of chronic obstructive pulmonary disease in the Southern Cone of Latin America: how different spirometric criteria may affect disease burden and health policies
Source: BMC Pulm Med. 2017 Dec 11;17:187. doi: 10.1186/s12890-017-0537-9 (PMC5725644; doi:10.1186/s12890-017-0537-9)
Supplement: Supplementary file 4 — Performance of the Fixed-Ratio method versus the Lower-Limit-of-Normal method. (DOCX 15 kb) [file 12890_2017_537_MOESM4_ESM.docx]

Supplementary Table 2. Performance of the Fixed-Ratio method versus the Lower-Limit-of-Normal method

|  | **COPD Prevalence** | | **Agreement (Kappa)** | **Sensibility** | **Specificity** | **Predictive**  **Value of**  **Positive Test** | **Predictive**  **Value of**  **Negative Test** |
| --- | --- | --- | --- | --- | --- | --- | --- |
|  | **According to FEV_1_/FVC** | **According to LLN** |  |  |  |  |  |
| Overall | 9.3 (8.4, 10.2) | 4.7 (4.1, 5.3) | 0.66 (0.65, 0.67) | 100.0 | 95.5 (95.4, 95.7) | 51.6 (50.5, 52.8) | 100.0 |
| Age groups, years |  |  |  |  |  |  |  |
| 45-54 | 4.8 (3.6, 5.9) | 3.5 (2.5, 4.4) | 0.84 (0.82, 0.85) | 100.0 | 98.7 (98.5, 98.8) | 72.7 (70.8, 74.7) | 100.0 |
| 55-64 | 9.5 (7.9, 11) | 4.8 (3.7, 5.9) | 0.65 (0.63, 0.67) | 100.0 | 95.1 (94.8, 95.3) | 50.5 (48.6, 52.4) | 100.0 |
| 65-74 | 17.9 (15.5, 20.3) | 7.0 (5.5, 8.5) | 0.51 (0.49, 0.53) | 100.0 | 88.2 (87.7, 88.8) | 38.9 (37.1, 40.6) | 100.0 |

Data are percentages (95% CI)
