# Supplementary material for: Estimating prevalence of chronic obstructive pulmonary disease in the Southern Cone of Latin America: how different spirometric criteria may affect disease burden and health policies
Source: BMC Pulm Med. 2017 Dec 11;17:187. doi: 10.1186/s12890-017-0537-9 (PMC5725644; doi:10.1186/s12890-017-0537-9)
Supplement: Supplementary file 5 — Age-standardized Prevalence (95% Confidence Intervals) of Chronic Obstructive Pulmonary Disease According to Demographic and Other Risk Factors. (DOCX 19 kb) [file 12890_2017_537_MOESM5_ESM.docx]

|  | **COPD according FEV_1_/FEVC** | | | | | **COPD according LLN** | | | | |  |  |
| --- | --- | --- | --- | --- | --- | --- | --- | --- | --- | --- | --- | --- |
|  | **Total** | **Men** | | **Women** | | **Total** | **Men** | | **Women** | |  |  |
| Education level |  |  | |  | |  |  | |  | |  |  |
| <High School | 10.8 (9.6, 12.0) | 13.1 (11.2, 15.1) | | 9.1 (7.6, 10.6) | | 5.9 (5.0, 6.8) | 6.8 (5.3, 8.2) | | 5.3 (4.1, 6.5) | |  |  |
| ≥High School | 6.9 (5.6, 8.3) | 9.9 (7.5, 12.3) | | 4.4 (3.0, 5.9) | | 2.9 (2.0, 3.8) | 4.4 (2.7, 6.0) | | 1.7 (0.8, 2.5) | |  |  |
| Body mass index |  |  | |  | |  |  | |  | |  |  |
| <25 kg/m^2^ | 12.7 (10.5, 14.9) | 17.1 (13.3, 21.0) | | 9.2 (6.7, 11.6) | | 7.9 (6.2, 9.7) | 10.5 (7.3, 13.7) | | 5.9 (4.1, 7.8) | |  |  |
| ≥25 kg/m^2^ | 8.4 (7.5, 9.4) | 10.5 (8.9, 12.1) | | 6.7 (5.6, 7.9) | | 3.9 (3.2, 4.5) | 4.6 (3.5, 5.7) | | 3.3 (2.4, 4.2) | |  |  |
| Cigarette smoking |  |  | |  | |  |  | |  | |  |  |
| Current smoker | 15.6 (13.2, 18.0) | 17.7 (14.0, 21.3) | | 13.6 (10.4, 16.8) | | 8.8 (7.0, 10.6) | 10.5 (7.6, 13.5) | | 7.0 (4.9, 9.1) | |  |  |
| Former smoker | 8.9 (7.3, 10.5) | 10.8 (8.5, 13.1) | | 6.3 (4.1, 8.4) | | 4.5 (3.3, 5.6) | 5.1 (3.5, 6.7) | | 3.6 (1.9, 5.3) | |  |  |
| Never smoker | 6.4 (5.3, 7.5) | 8.0 (6.0, 10.1) | | 5.7 (4.4, 7.0) | | 2.9 (2.1, 3.7) | 3.1 (1.7, 4.4) | | 2.9 (1.8, 3.9) | |  |  |
| Lifetime exposure in current smokers |  |  | |  | |  |  | |  | |  |  |
| 0-10 pack-years | 7.1 (3.2, 11.0) | 12.0 (4.7, 19.3) | | 2.9 (0.6, 5.1) | | 4.6 (1.9, 7.3) | 7.1 (1.7, 12.6) | | 2.5 (0.7, 4.3) | |  |  |
| 10-19 pack-years | 14.0 (8.4, 19.7) | 12.2 (4.9, 19.5) | | 18.2 (9.6, 26.8) | | 6.1 (2.4, 9.8) | 7.5 (1.4, 13.7) | | 5.8 (0.7, 10.9) | |  |  |
| ≥20 pack-years | 24.6 (20.4, 28.8) | 25.4 (19.6, 31.2) | | 23.6 (17.7, 29.5) | | 14.3 (11.0, 17.6) | 14.9 (10.1, 19.7) | | 14.0 (9.4, 18.6) | |  |  |
| Second-hand smoking |  |  | |  | |  |  | |  | |  |  |
| No | 8.3 (7.3, 9.2) | 10.2 (8.7, 11.8) | | 6.7 (5.6, 7.9) | | 4.2 (3.5, 4.9) | 4.9 (3.8, 6.0) | | 3.7 (2.8, 4.6) | |  |  |
| Yes | 14.6 (12.1, 17.1) | 19.6 (15.1, 24.0) | | 10.5 (7.9, 13.0) | | 7.6 (5.8, 9.4) | 10.7 (7.3, 14.0) | | 5.2 (3.4, 7.0) | |  |  |
| Exposure to biomass |  | |  | |  |  | |  | |  |  |  |
| No | 9.3 (8.3, 10.2) | | 11.2 (9.6, 12.8) | | 7.7 (6.5, 8.9) | 4.8 (4.1, 5.5) | | 5.6 (4.4, 6.7) | | 4.1 (3.2, 5.0) |  |  |
| Yes | 9.3 (7.0, 11.7) | | 13.4 (9.1, 17.6) | | 6.3 (3.8, 8.9) | 4.7 (2.9, 6.4) | | 6.4 (3.4, 9.5) | | 3.4 (1.4, 5.4) |  |  |
| Self-reported history of asthma |  | |  | |  |  | |  | |  |  |  |
| No | 7.7 (6.8, 8.5) | | 10.1 (8.7, 11.5) | | 5.7 (4.8, 6.6) | 3.4 (2.9, 4.0) | | 4.5 (3.5, 5.4) | | 2.6 (2.0, 3.3) |  |  |
| Yes | 34.0 (27.6, 40.4) | | 48.9 (36.4, 61.3) | | 27.7 (20.5, 35.0) | 24.0 (18.2, 29.8) | | 33.8 (22.1, 45.4) | | 19.9 (13.3, 26.5) |  |  |
| Self-reported history of tuberculosis |  | |  | |  |  | |  | |  |  |  |
| No | 9.0 (8.1, 9.9) | | 11.5 (10.0, 12.9) | | 7.1 (6.1, 8.1) | 4.4 (3.8, 5.0) | | 5.5 (4.4, 6.5) | | 3.6 (2.9, 4.4) |  |  |
| Yes | 24.9 (12.3, 37.6) | | 26.8 (10.4, 43.3) | | 24.6 (9.7, 39.6) | 21.6 (9.3, 33.8) | | 20.2 (5.4, 35.0) | | 23.0 (8.4, 37.7) |  |  |

Supplementary Table 3. Age-standardized Prevalence (95% Confidence Intervals) of Chronic Obstructive Pulmonary Disease According to Demographic and Other Risk Factors

Data are percentages (95% CI)

*Second-hand smoking was defined as exposed to second-hand smoking ≥5 days/week at home or work; biomass exposure was defined as the use of wood or coal for cooking ≥200 times/year
