# Supplementary material for: Estimating prevalence of chronic obstructive pulmonary disease in the Southern Cone of Latin America: how different spirometric criteria may affect disease burden and health policies
Source: BMC Pulm Med. 2017 Dec 11;17:187. doi: 10.1186/s12890-017-0537-9 (PMC5725644; doi:10.1186/s12890-017-0537-9)
Supplement: Supplementary file 6 — Age-standardized Prevalence (95% Confidence Intervals) of Chronic Obstructive Pulmonary Disease in Current Smokers according to intensity and duration exposure. (DOCX 14 kb) [file 12890_2017_537_MOESM6_ESM.docx]

Supplementary Table 4. Age-standardized Prevalence (95% Confidence Intervals) of Chronic Obstructive Pulmonary Disease in Current Smokers according to intensity and duration exposure

|  | **COPD according to FEV_1_/FVC** | | | | **COPD according to LLN** | | | |  |  |  |
| --- | --- | --- | --- | --- | --- | --- | --- | --- | --- | --- | --- |
|  | **Total** | **Stage I** | **Stage II** | **Stage III-IV** | **Total** | **Stage I** | **Stage II** | **Stage III-IV** |  |  |  |
| **No. of cigarettes per day** |  |  |  |  |  |  |  |  |  |  |  |
| 0 – 9 | 8.8 ( 5.8, 11.8) | 2.5 ( 0.8, 4.2) | 4.5 ( 2.3, 6.7) | 1.8 ( 0.4, 3.3) | 4.7 ( 2.6, 6.7) | 1.1 ( 0.1, 2.1) | 2.6 ( 0.9, 4.2) | 1.0 ( 0.2, 1.8) |  |  |  |
| 10 – 19 | 17.9 (12.9, 22.8) | 6.4 ( 2.8, 9.9) | 9.4 ( 5.7, 13.1) | 2.1 ( 0.1, 4.1) | 9.6 ( 5.8, 13.3) | 1.1 (0.0, 2.7) | 6.1 ( 3.1, 9.1) | 2.1 ( 0.1, 4.1) |  |  |  |
| ≥ 20 | 26.4 (20.8, 31.9) | 9.7 ( 5.6, 13.8) | 13.2 ( 9.3, 17.1) | 3.4 ( 1.1, 5.8) | 15.4 (11.0, 19.8) | 3.4 ( 0.9, 5.9) | 8.6 ( 5.5, 11.7) | 3.4 ( 1.1, 5.8) |  |  |  |
| **Time of exposure, years** |  |  |  |  |  |  |  |  |  |  |  |
| 0 - 19 | 2.7 (0.0, 6.0) | 0.8 (0.0, 2.3) | 1.5 (0.0, 4.5) | 0.3 (0.0, 1.0) | 1.9 (0.0, 4.9) | 0.0 | 1.5 (0.0, 4.5) | 0.3 (0.0, 1.0) |  |  |  |
| 20 – 40 | 11.3 ( 6.9, 15.6) | 3.8 ( 1.8, 5.7) | 6.3 ( 2.4, 10.1) | 1.2 ( 0.3, 2.1) | 4.8 ( 3.0, 6.5) | 1.6 ( 0.3, 2.8) | 2.0 ( 1.1, 2.9) | 1.2 ( 0.3, 2.1) |  |  |  |
| ≥ 40 | 23.0 (16.1, 30.0) | 7.5 ( 3.1, 12.0) | 11.8 ( 6.9, 16.7) | 3.7 ( 0.4, 6.9) | 16.5 ( 9.8, 23.2) | 4.5 ( 0.3, 8.7) | 8.8 ( 4.1, 13.5) | 3.2 (0.0, 6.3) |  |  |  |

Data are percentages (95% CI)
